# Effects of *in ovo* feeding of methionine and/or disaccharide on post-hatching breast development, glycogen reserves, nutrients absorption parameters, and jejunum antioxidant indices in geese

**DOI:** 10.3389/fvets.2022.944063

**Published:** 2022-08-22

**Authors:** De Xin Dang, Haizhu Zhou, Yujie Lou, Desheng Li

**Affiliations:** ^1^College of Animal Science and Veterinary Medicine, Jinzhou Medical University, Jinzhou, China; ^2^Department of Animal Resources Science, Dankook University, Cheonan, South Korea; ^3^College of Animal Science and Technology, Jilin Agricultural University, Changchun, China

**Keywords:** goose, *in ovo* injection, intestinal health, post-hatching development, nutrient absorption

## Abstract

We investigated the effects of *in ovo* injection of methionine (Met) and/or disaccharide (DS) on breast muscle and small intestine development, and the aspect of the glycogen contents, digestive enzymes activities, and jejunal antioxidant parameters in geese after incubation. A total of 600 fertilized eggs were used in this study to be employed in a 2 × 2 factorial experiment. Eggs were randomly assigned to 4 groups, 6 replicates per group, and 25 eggs per replicate. Factors in four groups included non-injection, Met injection (5 g/L Met dissolved in 7.5 g/L NaCl), DS injection (25 g/L maltose and 25 g/L sucrose dissolved in 7.5 g/L NaCl), and DS plus Met injection (25 g/L maltose, 25 g/L sucrose, and 5 g/L Met dissolved in 7.5 g/L NaCl). As a result, birth weight, relative weight of breast muscle, diameter of myofiber, glycogen contents, jejunal villus and surface area, and jejunal digestive enzymes activities improved, while liver glucose-6-phosphatase activity decreased, by DS injection. Additionally, DS administration upregulated the expression of myogenic factor-5 (Myf-5) from breast muscle and sodium/glucose cotransporter protein-1 (SGLT-1) from jejunum. *In ovo* delivery of DS has long-term effects on the improvement of jejunal glucose transporter-2 (GLUT-2) and sucrase-isomaltase expression. *In ovo* feeding of Met improved the relative weight of breast muscle and small intestine, diameter of myofiber, length of small intestine, jejunal villus width, jejunal sucrase, Na^+^/K^+^ATPase and alkaline phosphatase activities, and jejunal glutathione (GSH) concentration, and decreased the jejunal glutathione disulfide (GSSH) and the ratio of GSSG to GSH, in early-life post-hatching. The breast muscle Myf-5 and myostatin expression, jejunal villus height and surface area, jejunal glutathione peroxidase concentration, and the expression of GLUT-2 in jejunum long-term improved by *in ovo* delivery of Met. Moreover, *in ovo* feeding of DS plus Met mixture synergistically improved the diameter of myofiber, jejunal villus height and width, jejunal sucrase, and alkaline phosphatase activities in early-life post-hatching, but long-term upregulated the expression of jejunal GLUT-2. Therefore, we concluded that *in ovo* injection of Met plus DS is an effective way to improve the development of gosling during post-hatching stages.

## Introduction

Geese are seasonal egg producers, its eggs are mainly used for eating, reproduction, and research purposes. It becomes more important to improve gosling quality during post-hatching stages in commercial geese farming. In modern gosling hatcheries, birds are always taken out of the incubator at the same time, which means premature birds have to wait for other late-born birds to ensure maximum hatching over the same period. Additionally, commercially hatched gosling faces entirely different post-natal environments in comparison with geese in natural brooding; they could not have immediate access to feed and water. A series of operations such as packaging and shipping also inevitably delays the access to feed and water. Therefore, gosling faces a fasting challenge during its initial crucial period of life.

The vigorous growth and metabolism activities during the fasting periods lead to an insufficient supply of energy and protein and the risk of oxidative stress, resulting in retarded growth, limited gut development, and stunted breast muscle (1–4). The *in ovo* injection approach provides a method in overcoming these problems and filling the gap between the hatch and first feed access (5).

Amnion has been proven to be an effective site for implementing *in ovo* injection techniques (6). The embryo orally consumes the amniotic fluid before pipping the air cell under natural conditions, thus consuming the supplemented nutrients which are presented in the enteric tissues (7). This presents an opportunity to inject nutrients into the amnion to uplift the status of growth and development in the embryo (8).

Digestible disaccharides (DS) are possible candidates for exogenous energy provision, it plays an important glucose precursor for late-term bird embryo catabolism and could alleviate energy deficiency by stimulating glucose anabolism (9, 10). Methionine (Met) as the first limiting amino acid for birds has been reported to decrease oxidative stress and uplift the protein supply (11, 12). To our knowledge, studies on the effects of *in ovo* injection of DS and/or Met on the performance in geese were still limited. In our previous study, *in ovo* injection of DS and/or Met had positive effects on the development of the embryo of goslings (13). We hypothesized that the administration of DS and/or Met into the amnion of the late-term embryo may serve as a tool to provide energy for small intestine and embryo activity, in turn alleviating energy and protein lack, and improving breast muscle and intestine development in geese during post-hatching periods. To test this hypothesis, we conducted this study to investigate the effects of *in ovo* injection of DS and/or Met on the post-hatching breast muscle parameters, glycogen reserves, glucose-6-phosphatase (G-6-Pase) activity, myogenic factor-5 (Myf-5) and myostatin (MSTN) expression in breast muscle, small intestine parameters, jejunum morphology, jejunum digestive enzymes activities, jejunum antioxidant indices, jejunum sodium/glucose cotransporter protein-1 (SGLT-1), glucose transporter-2 (GLUT-2), and sucrase-isomaltase (SI) expression.

## Materials and methods

### Experimental design, animals, and housing

The fertilized eggs (in total 1,000) were purchased from the Dekun Poultry Food Co., Ltd (Meihekou, Jilin). The breed of the egg was Jilin White geese. All eggs were laid on the same day, represented in the same weight class. Fertilized geese eggs were incubated in the standard condition in an incubator (Keyu CFZ microcomputer automatic incubator, Dezhou, Shandong). Before transiting to the incubator, eggs were pre-heated to 30°C for 12 h, disinfected with 37% formaldehyde and potassium permanganate (2:1), and distributed into incubator tray levels. The incubation period included three phases (phase 1, days 1–14; phase 2, days 15–28, and phase 3, days 29–31). During phase 1, the temperature was 38°C and the humidity was 65%; during phase 2, the temperature was 37.5°C and the humidity was 55%; and during phase 3, the temperature was 37.2°C and the humidity was 70%. All eggs were turned once per 2 h for 180 s.

Eggs were candled checking for containing embryonated eggs on day 23 of incubation. A total of 600 fertilized eggs were selected from the embryonated eggs obtained above and randomly assigned into 4 groups with 6 replicates per group and 25 eggs per replicate. This experiment was a two-factor design, which included DS injection or Met injection. Eggs treatment in four groups was divided into non-injection, Met injection (5 g/L Met dissolved in 7.5 g/L NaCl), DS injection (25 g/L maltose and 25 g/L sucrose dissolved in 7.5 g/L NaCl), and DS plus Met injection (25 g/L maltose, 25 g/L sucrose, and 5 g/L Met dissolved in 7.5 g/L NaCl). Injection solutions and paraffin were prepared on the day of injection (day 24 of incubation) and were treated at 121°C for 15 min. The injection was conducted when the solutions and paraffin are cooled down to room temperature.

A 70% of ethanol was used for egg surface sterilization. Cleaned eggs were put onto a holder with the large end on top. The position of amnion was identified by candling. The upper side of the eggs (the air space) was pierced by an egg-shell punch. Injections were performed with a disinfected injector. About 1.5 mL of each solution was injected into the amnion of each egg to a depth of 20 mm, without hurting the embryo. After each inoculation, the needle was routinely disinfected to minimize the risk of infection. All eggs were held outside the incubator for <5 min while injecting, including the non-injected control eggs. Immediately after the injection, the hole was sealed using paraffin, and the eggs were returned to the incubator and incubated in line with the routine procedure until hatched.

After hatching, birds were transported to a temperature-controlled room with continuous lighting and distributed into cages according to the replicates. Feeds were provided immediately after transportation. The raising period was until day 28 of age (Table 1). Uniform management was conducted during this period. The temperature of the room was maintained at 30°C during the first 3 days and then reduced by 2°C per week.

**Table 1:** Composition and nutrient levels of the experimental basal diet (%, as-fed basis).

**Ingredients, %**	
Corn	60.00
Soybean meal	29.11
Wheat bran	6.00
Fish meal	2.00
Lysine-HCl	0.20
Methionine	0.23
Dicalcium phosphate	0.84
Limestone	0.82
Sodium chloride	0.30
Vitamin and trace mineral premix^a^	0.50
Total	100.00
Calculated value, %	
Metabolizable energy, MJ/kg	11.67
Available phosphorus	0.40
Analyzed composition, %	
Crude protein	19.78
Methionine	0.50
Total sulfate amino acid	0.77
Lysine	1.08
Calcium	0.78
Crude fiber	0.31
Neutral detergent fiber	1.09
Acid detergent fiber	0.35

a Provided per kg of complete diet: vitamin D_3_, 200 IU; vitamin A (retinyl acetate), 1,500 mg; vitamin E (DL-α-tocopheryl acetate), 12.5 mg; vitamin K_3_, 1.5 mg; thiamine, 2.2 mg; riboflavin, 5 mg; nicotinic acid, 65 mg; folic acid, 1 mg; pantothenic acid, 15 mg; pyridoxine, 2 mg; biotin, 0.2 mg; choline, 1,000 mg; Fe, 90 mg; Cu, 6 mg; Mn, 85 mg; Zn, 85 mg; I, 0.42 mg; Se, 0.3 mg; Co, 2.5 mg.

The Animal Care and Use Committee of Jilin Agricultural University (Changchun, China) supervised the procedure of this experiment.

### Feed analysis

After homogeneous mixing, feed samples were collected from each dietary group. All feed samples were dried in a 70°C constant temperature oven for 72 h. Subsequently, feed samples were ground and sieved with a 1-mm sieve. The collected feed powder is with a diameter of <1 mm for feed composition analysis. According to the procedure established by the AOAC (14), the dry matter (method 930.15), crude protein (nitrogen × 6.25; method 968.06), and crude fiber (method 991.43) composition in the diet were analyzed. Then, the representative feed samples in each group were hydrolyzed with 6 N HCl for 24 h at 110°C. An amino acid analyzer (2690 Alliance, Waters, Inc., Milford, MA) was used for determining amino acid contents in the diet. In addition, the contents of neutral detergent fiber and acid detergent fiber in the diet were measured according to the method provided by Mertens (15).

### Sample collection

On hatching day, day 7 post-hatching, and day 28 post-hatching, three birds were randomly selected from each replicate group, weighed, and slaughtered by cervical dislocation for further analysis.

The liver was removed from the carcass and the adherent material of the liver was carefully removed under ice-cold saline. About 100 mg of liver samples were taken and homogenized in 0.25 mol l^−1^ sucrose solution for G-6-Pase activity analysis. The remaining sample of the liver was frozen as aliquots in liquid nitrogen, and stored at −80°C for measuring the liver glycogen contents.

Both sides of breast muscle were stripped after being slaughtered. One side of the breast muscle sample was weighed and fixed with 10% neutral-buffered formalin for analyzing myofiber traits. Another side of the breast muscle sample was frozen as aliquots in liquid nitrogen, and stored at −80°C for measuring breast muscle glycogen contents and breast muscle development-related gene expression situation.

The whole small intestine was removed and the adherent material of the small intestine was carefully removed under ice-cold saline, weighed, and separated into the duodenum, jejunum, and ileum. The duodenum was defined as the portion of the small intestine composed of the duodenal loop; the jejunum was considered as the part between the duodenum and ileum; and the ileum as a distal segment before ileo-cecal junction equal to the length of the cecum. The jejunum sample was used for measuring antioxidant parameters first, and then about 1-cm long segment from the middle of the jejunum was taken in duplicate and placed in two separate tubes. One sample was fixed with 10% neutral-buffered formalin solution for histology and the other sample was frozen in liquid nitrogen, and then stored at −80°C for measuring digestive enzyme activities and nutrients transport gene expression.

### Experimental parameters measurement

#### Growth performance parameters analysis

The body weight of goslings was checked after incubation to calculate the birth weight and correspondingly calculated the mortality rate.

#### Breast muscle and small intestine parameters analysis

The relative weight of breast muscle and small intestine were calculated using the following equation:


$$\begin{array}{l} Organ\;index\;=\;\frac{Organ\;weight}{Live\;body\;weight}\;\times \;100\;\%. \end{array}$$

The length of the small intestine was measured by a dividing ruler.

#### Myofiber traits analysis

After slaughtering the geese, the breast muscle samples were cut into small pieces and fixed with 10% neutral buffered formalin for 12 h, followed by dehydration in increasing concentrations of alcohol (70, 80, 90, 95, and 100%) and xylene. Consequently, samples were embedded in paraffin and stored in an oven at 60°C. About 12 h later, samples were removed from the oven and histological cassettes. Fragments were placed in “paper boxes” and covered with paraffin. After the paraffin solidified into blocks, the “papers” were removed and the blocks were kept under refrigeration until the cuts were realized (16).

Serial tissue sections (3 μm thickness) were excised perpendicular to the direction of the myofibers using a cryostat. After sectioning, put the paraffin section ribbon on the coating slide glass. Dried slides were kept in an oven at 60°C for 2 h to eliminate any excess paraffin. The next step consisted of paraffin removal and slide hydration, using xylene, and different concentrations of ethanol. Samples were then stained following the hematoxylin and eosin staining protocol (16).

Samples were then dehydrated again and mounted. In each specimen, the diameters of muscle fibers were measured under a light microscope equipped with a ScopePhpto (LY-WN 300, Hangzhou Scopetek Opto-Eletric Co., Ltd.).

No less than 150 intact, well-oriented muscle fibers cross-sectional area of five fields of vision were measured under 40 times the objective lens. With muscle fibers assumed to be round, the muscle fiber cross-sectional area (A) was converted to diameter (D) by the formula, D = 2√A/π. The average value was calculated to represent the diameter of the muscle fibers (17).

#### Glycogen reserves analysis

About 0.1 grams sample of liver and breast muscle were stored at 1 ml of 8% HClO4, homogenized (in ice) for 45 s, and centrifuged at 7,700 rpm at 4°C for 16 min. A 10-μl aliquot of the supernatant was transferred to a clean polypropylene tube, along with 0.4 ml of 8% perchloric acid and 2.6 ml of iodine color reagent made of 1.3 ml of solution A (0.26 g iodine + 2.6 g potassium iodide dissolved in 10 ml of distilled water) in 100 ml of 67.8% saturated calcium chloride. All samples were read at a wavelength of 450 nm. The amount of glycogen present in the sample solution was determined by the preparation of a known glycogen standard curve.

#### G-6-Pase activity analysis

Livers were taken to a total weight of 100 mg and homogenized in 0.25 mol l^−1^ sucrose solution for analyzing G-6-Pase activity. G-6-Pase was assayed (in 20 mmol l^−1^ Tris–HCl, pH 7.3 for 10 min at 37°C) by complex formation of inorganic phosphate (Pi) liberated from G-6-Pase (0.1 mol l^−1^) and subtracting the amount of Pi produced from para-nitrophenylphosphate (20 mmol l^−1^) under the same conditions of the assay. The protein concentration in liver homogenization was assayed using the method described by Bradford (18). G-6-Pase activity was expressed in micromoles of released inorganic phosphorus per min per milligram protein.

#### Muscle growth-related gene expression analysis

Total RNA was isolated from muscle samples using RNAiso Reagent (TaKaRa, Dalian, Liaoning, China). The RNA integrity was assessed by electrophoresis on a 1% agarose gel containing formaldehyde. The RNA concentration was measured using a Beckman DU-640 spectrophotometer (Beckman). The sequences of primers for the genes tested were specifically designed according to the sequences located in GenBank (Table 2). The total RNA samples were purified and subjected to reverse transcription using the Takara PrimeScript RT Reagent Kit with gDNA Eraser (Takara, Dalian, China) and processed for cDNA synthesis as per Takara PrimeScript RT instructions (19).

**Table 2:** Primers used for quantitative real-time PCR.

**Gene**	**Accession^a^**	**Product**		**Primer sequences**	
		**(bp)^b^**		
β-Actin	M26111	158	Forward	GCCCAGCACGATGAAGAT
			Reverse	ATTTACGGTGGACGATGGAC
MSTN	AY448009	133	Forward	GTGGCTCTTGATGACGGTAGT
			Reverse	GCAGTGTGCTGAGGATTTGA
Myf-5	KU744843	147	Forward	GCGTTTGAGACCCTGAAGAG
			Reverse	TCCCGGCAGGTGATAGTAGT
SGLT-1	KU744842	126	Forward	GTAACATTGGCAGCGGACAT
			Reverse	TGGGTACAAACAGCCATCCT
GLUT-2	KU744841	118	Forward	CAGTTCTTCCTGCTCCTGCT
			Reverse	TCATCGGGTCACAGTTTCCT
SI	KU744844	193	Forward	CGTCACCTTCCCTCTTTGG
			Reverse	GGATTATGCTTCACTTCCACTTTG

SGLT-1, sodium/glucose cotransporter protein-1; SI, sucrase-isomaltase; Myf-5, myogenic factor-5; GLUT2, glucose transporter-2; MSTN, myostatin.

a Accession number refer to Genbank (NCBI).

b PCR product size (base pairs).

The relative expression levels of Myf-5 and MSTN genes in pectoral muscle were analyzed by RT-PCR, which was performed in a 10-μL reaction mix containing 1 μL 2 × SYBR Premix Ex Taq II (TakaRa, Dalian, China), 3 μL dH_2_O, 0.5 μL of the upstream and downstream primers, and 1 μL cDNA using a Bio-Rad CFX-96 thermocycler (Bio-Rad, CA). The reaction conditions were as follows: initial denaturation at 95°C for 30 s and 44 cycles of amplification at 72°C for 30 s. The annealing was carried out for 40 s at temperatures specific to each target gene. At the end of the amplification, step-wise melting curves were performed to confirm the product specificity. The cytoskeletal protein, β-actin, was used as the internal reference (20). All the reactions were performed in triplicate. The qRT-PCR data were analyzed using the 2^−ΔΔCt^ method. The relative level of each mRNA normalized to the ΔCt (β-actin) gene was calculated using the following equation:


$$\begin{array}{l} Fold\;change\\ =\frac{2^{Ct\;target\;gene\;\left(control\right)\;-Ct\;target\;gene\;\left(treatment\right)}}{2^{Ct\;housekeeping\;gene\;\left(control\right)-Ct\;housekeeping\;gene\;\left(treatment\right)}} \end{array}$$

#### Jejunum morphology analysis

The jejunum samples were cut into small pieces and further treated by fix and dehydration according to the methods described above to prepare paraffin blocks.

A microtome was used to make five cuts that were 5 μm. The cuts were stained with hematoxylin-eosin. The values were measured using a light microscope. Measurements of villus height and width were determined at a magnification of 10X. A minimum of five measurements per slide were made for each parameter and averaged into one value (21). Villus surface area was calculated from the villus height (from the tip of the villi to the villus crypt junction) and width at half height (22). Values presented means from 10 adjacent villi and only vertically oriented villi were measured.

#### Digestive enzymes activities analysis

Enzyme activities were assayed in homogenized jejunum tissue. Samples were thawed at 4°C and homogenized in 10 times the volume of cold normal saline. The homogenates were then centrifuged at 20,000 ×g for 20 min at 4°C and the supernatant was collected for enzyme assays. Sucrase [Enzyme Commission (EC) 3.2.1.48] and maltase (EC 3.2.1.20) activity were assayed colorimetrically using sucrose and maltose as substrates, respectively. The activity was expressed as micromoles of glucose released per minute per gram of jejunum wet tissue. Alkaline phosphatase (EC 3.1.3.1) activity was determined by measuring the hydrolysis of p-nitrophenol at 37°C, and the unit of activity was expressed as per min per gram of jejunum wet tissue. Na^+^/K^+^ATPase (EC 3.6.1.3) activity was determined by measuring the liberation of phosphate from ATP-Na2 (No. A7699, Sigma-Aldrich) in two media: medium I (all ATPases system) and medium II (Na^+^/K^+^ATPase restrained system); and the activity of Na^+^/K^+^ATPase was calculated as the difference between phosphates liberated by each homogenate in the two media and was expressed as micromoles of phosphates per milligram homogenate protein or per milliliter of serum per h.

#### Jejunum antioxidant parameters analysis

The activities of glutathione peroxidase (GPX), glutathione (GSH), and glutathione disulfide (GSSG) in the mucose were determined using commercial kits (Nanjing JianCheng Bioengineering Institute, Nanjing, P. R. China) according to the instructions of the manufacturer. All results were normalized against total protein concentration in each sample for inter-sample comparison. The GSSG/GSH ratio was determined according to the data on GSSG and GSH activities in mucose.

#### Jejunum nutrients transport gene expression analysis

The abundance of SGLT-1, GLUT-2, and SI mRNA isolated from geese jejunum tissues (approximately 50 mg) was analyzed by the above step. The sequences of primers for the genes tested were specifically designed according to the sequences located in GenBank (Table 2). The cytoskeletal protein, β-actin, was used as the internal reference (20).

#### Statistical analysis

The data were analyzed as a two-way ANOVA factorial arrangement of treatments using the GLM procedure in SPSS18.0 software, with treatment as the fixed effect and the replicate cage as the experimental unit. Factors involved were *in ovo* injection of DS and/or Met. The data are presented as the means ± standard deviation (SD). Results were considered significant at *P* < 0.05.

## Results

### Growth performance parameter

Birth weight was increased by feeding DS into the embryo (*P* < 0.05). The *in ovo* injection strategies had no significant effects on the mortality rate (Table 3).

**Table 3:** Effects of *in ovo* injection of methionine (Met) and disaccharide (DS) on growth performance parameters of geese during the early-life post-hatching^a, b^.

**Met**	−		+		***P*** **-value**		
**DS**	**−**	**+**	**−**	**+**	**Met**	**DS**	**Met × DS**
Birth weight, g	95.93 ± 5.14	101.64 ± 7.27	99.35 ± 3.28	105.68 ± 9.98	0.199	0.045	0.912
Morality rate, %	3.47 ± 1.44	2.68 ± 2.70	3.65 ± 1.65	2.66 ± 0.04	0.937	0.399	0.919

a The data are presented as the means ± standard deviation.

b The “−” means without nutrients injection, while “+” means with nutrients injection.

### Breast muscle and myofiber trait parameter

*In ovo* injection of DS increased the relative weight of breast muscle (*P* < 0.01) and the diameter of myofiber (*P* < 0.01) on the day of hatching. Met administration increased the relative weight of breast muscle on the day of hatching (*P* < 0.01) and day 7 post-hatching (*P* < 0.05) and increased the diameter of myofiber on day 7 post-hatching (*P* < 0.01). In addition, *in ovo* delivery of Met plus DS synergistically increased the diameter of myofiber on the day of hatching (*P* < 0.01) (Table 4).

**Table 4:** Effects of *in ovo* injection of methionine (Met) and disaccharide (DS) on breast muscle parameters of geese during the early-life post-hatching^1, 2^.

**Met**	−		+		***P*** **-value**		
**DS**	**−**	**+**	**−**	**+**	**Met**	**DS**	**Met × DS**
Relative weight of breast muscle, %							
Day of hatching	0.67 ± 0.05	0.91 ± 0.13	0.92 ± 0.08	1.03 ± 0.06	<0.001	<0.001	0.059
Day 7 post-hatching	0.70 ± 0.02	0.71 ± 0.06	0.74 ± 0.09	0.79 ± 0.07	0.031	0.206	0.425
Day 28 post-hatching	1.31 ± 0.13	1.22 ± 0.16	1.34 ± 0.33	1.23 ± 0.22	0.830	0.280	0.945
Diameter of myofiber, μm							
Day of hatching	5.31 ± 0.09^c^	6.37 ± 0.36^a^	5.88 ± 0.36^b^	6.24 ± 0.26^a^	0.080	<0.001	0.007
Day 7 post-hatching	7.02 ± 0.32	7.02 ± 0.32	7.63 ± 0.84	7.96 ± 0.34	0.001	0.431	0.431
Day 28 post-hatching	12.55 ± 1.34	12.43 ± 0.49	12.93 ± 1.07	12.51 ± 1.04	0.586	0.524	0.720

1 The data are presented as the means ± standard deviation.

2 The “−” means without nutrients injection, while “+” means with nutrients injection.

a, b, c Different superscripts within a row indicate a significant difference (*p* < 0.05).

### Glycogen reserves

As shown in Table 5, *in ovo* injection of Met had no significant effects on the glycogen reserves. However, *in ovo* feeding of DS increased breast muscle glycogen content on the day of hatching (*P* < 0.01) and day 7 post-hatching (*P* < 0.01), liver glycogen content on the day of hatching (*P* < 0.01), and total glycogen content on the day of hatching (*P* < 0.01).

**Table 5:** Effects of *in ovo* injection of methionine (Met) and disaccharide (DS) on glycogen reserves of geese during the early-life post-hatching^a, b^.

**Met**	−		+		***P*** **-value**		
**DS**	**−**	**+**	**−**	**+**	**Met**	**DS**	**Met × DS**
Breast muscle glycogen contents, mg/g							
Day of hatching	1.73 ± 0.17	1.95 ± 0.09	1.73 ± 0.24	2.12 ± 0.17	0.274	<0.001	0.240
Day 7 post-hatching	2.00 ± 0.10	2.40 ± 0.20	2.14 ± 0.29	2.54 ± 0.17	0.112	<0.001	0.991
Day 28 post-hatching	1.53 ± 0.14	1.53 ± 0.09	1.61 ± 0.16	1.55 ± 0.22	0.478	0.690	0.681
Liver glycogen contents, mg/g							
Day of hatching	3.31 ± 0.29	4.08 ± 0.17	3.48 ± 0.51	4.21 ± 0.53	0.374	<0.001	0.926
Day 7 post-hatching	64.58 ± 4.48	63.96 ± 2.00	66.20 ± 4.42	65.34 ± 2.03	0.300	0.607	0.933
Day 28 post-hatching	43.77 ± 4.10	45.68 ± 3.12	46.16 ± 4.00	42.15 ± 3.06	0.699	0.484	0.058
Total glycogen contents, mg/g							
Day of hatching	0.10 ± 0.01	0.14 ± 0.02	0.12 ± 0.01	0.15 ± 0.03	0.110	<0.001	0.665
Day 7 post-hatching	2.90 ± 0.47	2.81 ± 0.38	2.92 ± 0.15	2.88 ± 0.21	0.727	0.642	0.890
Day 28 post-hatching	1.34 ± 0.14	1.29 ± 0.26	1.40 ± 0.20	1.19 ± 0.26	0.805	0.177	0.382

a The data are presented as the means ± standard deviation.

b The “−” means without nutrients injection, while “+” means with nutrients injection.

### G-6-Pase activity

DS injection decreased the G-6-Pase activity on the day of hatching (*P* < 0.01). However, Met injection had no significant effects on the G-6-Pase activity (Table 6).

**Table 6:** Effects of *in ovo* injection of methionine (Met) and disaccharide (DS) on glucose 6-phosphatase (G-6-Pase) activity of geese during the early-life post-hatching^a, b^.

**Met**	−		+		***P*** **-value**		
**DS**	**−**	**+**	**−**	**+**	**Met**	**DS**	**Met × DS**
G-6-Pase activity							
Day of hatching	0.17 ± 0.01	0.14 ± 0.01	0.15 ± 0.02	0.13 ± 0.01	0.085	<0.001	0.237
Day 7 post-hatching	0.03 ± 0.003	0.03 ± 0.004	0.03 ± 0.01	0.03 ± 0.01	0.990	0.498	0.467
Day 28 post-hatching	0.03 ± 0.01	0.03 ± 0.01	0.03 ± 0.01	0.03 ± 0.01	0.881	0.758	0.657

a The data are presented as the means ± standard deviation.

b The “−” means without nutrients injection, while “+” means with nutrients injection.

### Muscle growth-related gene expression

The expression of Myf-5 from breast muscle was upregulated by DS injection on the day of hatching (*P* < 0.05). Moreover, Met injection upregulated the Myf-5 and MSTN expression on the day of hatching (*P* < 0.01), day 7 post-hatching (*P* < 0.01), and day 28 post-hatching (*P* < 0.01) (Table 7).

**Table 7:** Effects of *in ovo* injection of methionine (Met) and disaccharide (DS) on the expression of myogenic factor-5 (Myf-5) and myostatin (MSTN) from breast muscle of geese during the early-life post-hatching^a, b^.

**Met**	−		+		***P*** **-value**		
**DS**	**−**	**+**	**−**	**+**	**Met**	**DS**	**Met × DS**
Myf-5 expression abundance in breast muscle							
Day of hatching	1.00 ± 0.19	1.19 ± 0.17	1.50 ± 0.17	1.61 ± 0.16	<0.001	0.048	0.553
Day 7 post-hatching	2.53 ± 0.27	2.55 ± 0.32	3.08 ± 0.24	3.03 ± 0.55	0.003	0.946	0.815
Day 28 post-hatching	4.61 ± 0.33	4.58 ± 0.73	5.87 ± 0.86	5.62 ± 0.64	<0.001	0.608	0.688
MSTN expression abundance in breast muscle							
Day of hatching	1.00 ± 0.22	1.11 ± 0.16	0.73 ± 0.08	0.64 ± 0.07	<0.001	0.692	0.066
Day 7 post-hatching	4.40 ± 0.62	4.39 ± 1.17	3.12 ± 0.63	2.92 ± 0.37	<0.001	0.737	0.768
Day 28 post-hatching	1.36 ± 0.17	1.40 ± 0.20	0.83 ± 0.11	0.77 ± 0.10	<0.001	0.894	0.430

a The data are presented as the means ± standard deviation.

b The “−” means without nutrients injection, while “+” means with nutrients injection.

### Small intestine parameter

Met injection increased the relative weight of the small intestine on the day of hatching (*P* < 0.05) and the length of the small intestine on day 7 post-hatching (*P* < 0.05). However, DS injection had no significant effects on the small intestine parameters (Table 8).

**Table 8:** Effects of *in ovo* injection of methionine (Met) and disaccharide (DS) on small intestine parameters of geese during the early-life post-hatching^a, b^.

**Met**	−		+		***P*-value**		
**DS**	**−**	**+**	**−**	**+**	**Met**	**DS**	**Met × DS**
Relative weight of small intestine, %							
Day of hatching	2.54 ± 0.12	2.37 ± 0.15	2.61 ± 0.23	2.64 ± 0.17	0.027	0.320	0.190
Day 7 post-hatching	7.69 ± 0.62	7.87 ± 0.29	7.93 ± 0.46	8.08 ± 0.34	0.234	0.373	0.929
Day 28 post-hatching	5.04 ± 0.41	5.21 ± 0.32	5.36 ± 0.33	5.34 ± 0.38	0.154	0.629	0.534
Length of small intestine, cm							
Day of hatching	48.48 ± 1.65	47.93 ± 0.61	49.25 ± 1.07	48.65 ± 1.82	0.201	0.317	0.965
Day 7 post-hatching	97.95 ± 1.60	97.72 ± 0.55	99.65 ± 2.99	101.40 ± 3.55	0.015	0.461	0.337
Day 28 post-hatching	154.17 ± 14.57	160.42 ± 16.29	156.33 ± 12.42	165.08 ± 11.15	0.550	0.196	0.826

a The data are presented as the means ± standard deviation.

b The “−” means without nutrients injection, while “+” means with nutrients injection.

### Jejunum morphology

**Figure 1:**
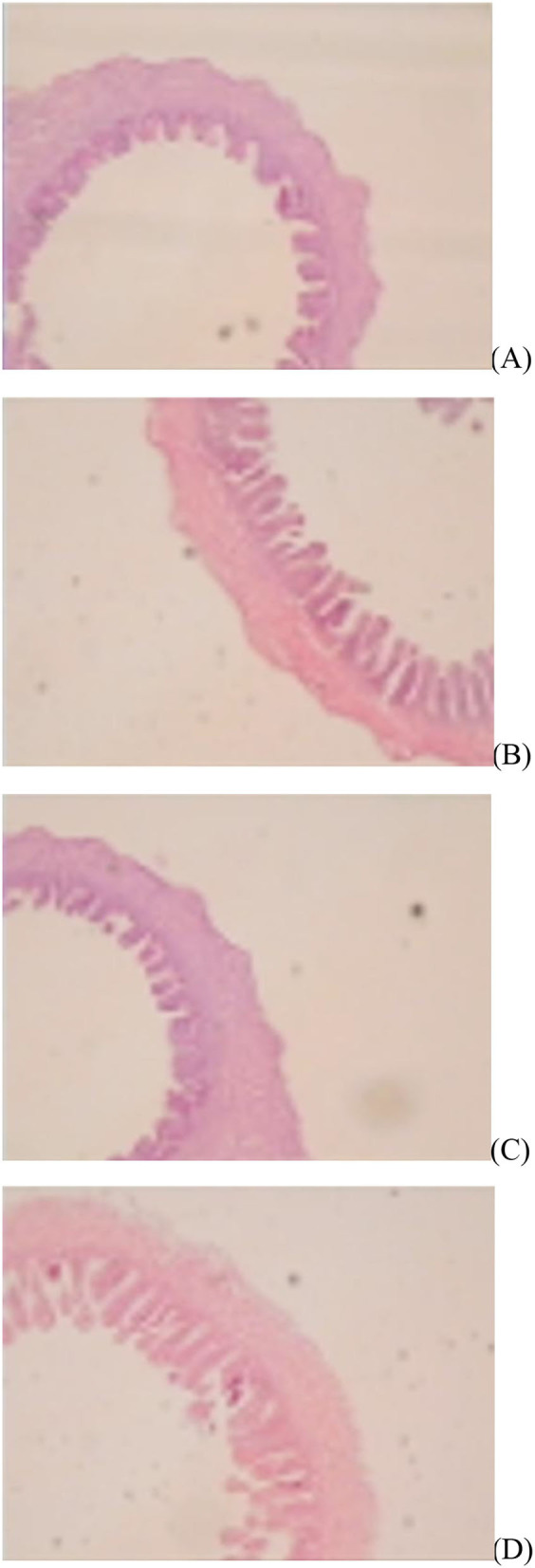
Slice of the jejunum in gosling treated by *in ovo* injection of methionine (Met) and/or disaccharide (DS) on day of hatching. **(A)** Non-injection group; **(B)** DS injection group; **(C)** Met injection group and **(D)** DS plus Met injection group.

**Figure 2:**
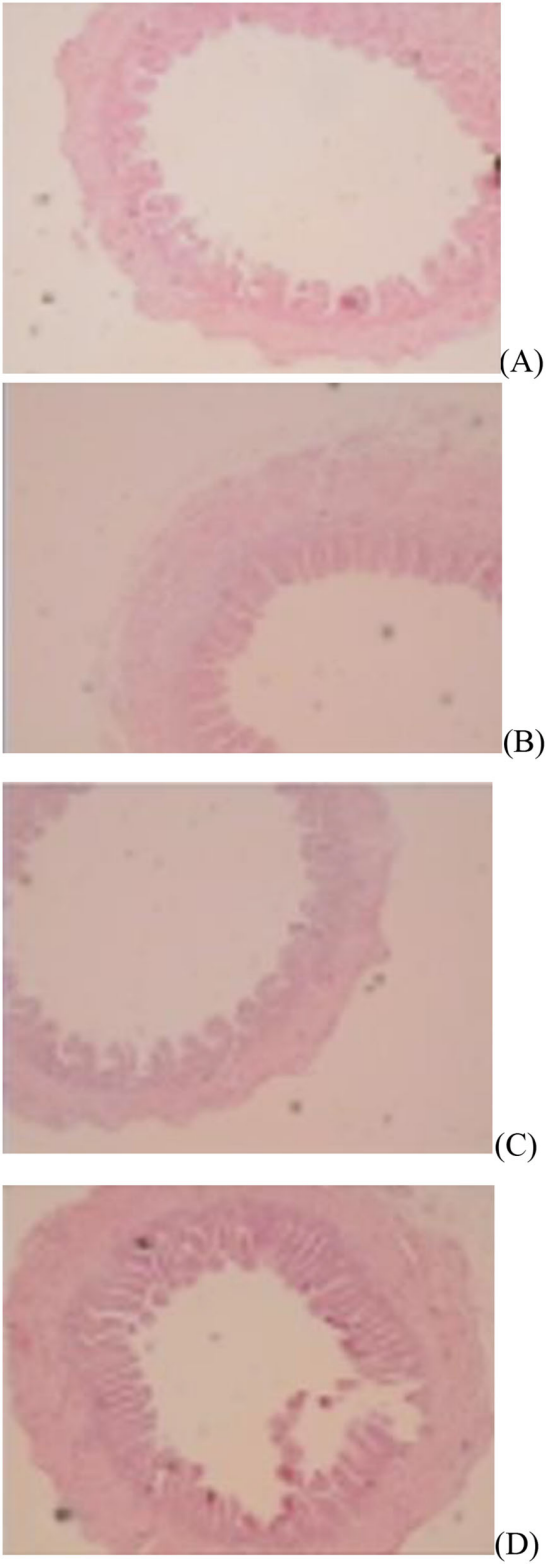
Slice of the jejunum in gosling treated by *in ovo* injection of methionine (Met) and/or disaccharide (DS) on day 7 post-hatching. **(A)** Non-injection group; **(B)** DS injection group; **(C)** Met injection group and **(D)** DS plus Met injection group.

**Figure 3:**
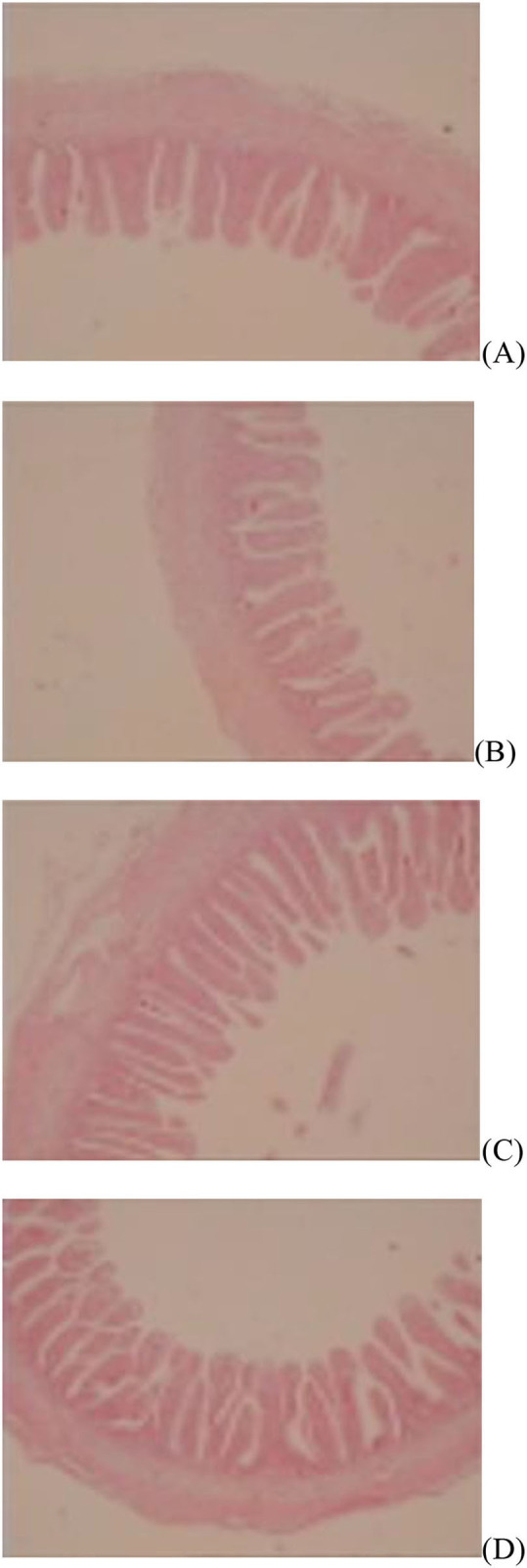
Slice of the jejunum in gosling treated by *in ovo* injection of methionine (Met) and/or disaccharide (DS) on day 28 post-hatching. **(A)** Non-injection group; **(B)** DS injection group; **(C)** Met injection group and **(D)** DS plus Met injection group.

The jejunal villus height (*P* < 0.01), villus width (*P* < 0.05), and villus surface area (*P* < 0.01) on day of hatching, villus height (*P* < 0.01) on day 7 post-hatching, and villus height (*P* < 0.01) and villus surface area (*P* < 0.01) on day 28 post-hatching were increased by *in ovo* injection of Met. DS injection increased jejunal villus height and villus surface area on the day of hatching (*P* < 0.01) and day 7 post-hatching (*P* < 0.01). Additionally, a synergistic effect of DS plus Met injection on the jejunal villus height (*P* < 0.01) and villus surface area (*P* < 0.01) was observed on the day of hatching (Table 9; Figures 1–3).

**Table 9:** Effects of *in ovo* injection of methionine (Met) and disaccharide (DS) on jejunum parameters of geese during the early-life post-hatching^1, 2^.

**Met**	−		+		***P*-value**		
**DS**	**−**	**+**	**−**	**+**	**Met**	**DS**	**Met × DS**
Villus height of jejunum, μm							
Day of hatching	138.56 ± 7.82^c^	219.82 ± 9.26^a^	194.89 ± 18.55^b^	221.72 ± 10.09^a^	<0.001	<0.001	<0.001
Day 7 post-hatching	471.42 ± 52.42	664.73 ± 23.02	556.17 ± 44.75	710.61 ± 56.49	0.002	<0.001	0.313
Day 28 post-hatching	1,030.49 ± 64.42	1056.30 ± 164.03	1173.26 ± 43.94	1190.12 ± 93.48	0.003	0.615	0.916
Villus width of jejunum, μm							
Day of hatching	38.84 ± 5.75^b^	35.82 ± 5.21^b^	36.59 ± 2.46^b^	46.84 ± 5.15^a^	0.037	0.081	0.003
Day 7 post-hatching	101.64 ± 13.01	108.22 ± 15.03	100.67 ± 12.57	108.94 ± 9.17	0.981	0.165	0.871
Day 28 post-hatching	192.77 ± 16.54	195.01 ± 10.27	208.45 ± 15.19	200.52 ± 13.92	0.082	0.628	0.390
Villus surface area of jejunum, μm^2^ × 10^3^							
Day of hatching	5.38 ± 0.85	7.91 ± 1.45	7.17 ± 1.11	10.35 ± 0.85	<0.001	<0.001	0.471
Day 7 post-hatching	47.48 ± 3.66	72.22 ± 12.69	55.54 ± 2.61	77.75 ± 11.59	0.076	<0.001	0.731
Day 28 post-hatching	198.24 ± 15.37	207.40 ± 43.92	245.13 ± 26.59	237.66 ± 8.88	0.002	0.940	0.462

1 The data are presented as the means ± standard deviation.

2 The “−” means without nutrients injection, while “+” means with nutrients injection.

a, b, c Different superscripts within a row indicate a significant difference (*p* < 0.05).

### Digestive enzymes activities

The Na^+^/K^+^ATPase (*P* < 0.05) and alkaline phosphatase (*P* < 0.01) activities on the day of hatching and sucrase (*P* < 0.01) and alkaline phosphatase (*P* < 0.01) activity on day 7 post-hatching were increased by *in ovo* Met delivery. *In ovo* feeding of DS increased maltase (*P* < 0.01), sucrase (*P* < 0.01), Na^+^/K^+^ATPase (*P* < 0.05), and alkaline phosphatase (*P* < 0.01) activities on the day of hatching, also increased sucrase (*P* < 0.01) and alkaline phosphatase (*P* < 0.01) activities on day 7 post-hatching. Additionally, *in ovo* injection of Met plus DS synergistically increased the sucrase activity (*P* < 0.01) on day 7 post-hatching and the alkaline phosphatase activity (*P* < 0.01) on the day of hatching (Table 10).

**Table 10:** Effects of *in ovo* injection of methionine (Met) and disaccharide (DS) on jejunum digestive enzymes parameters of geese during the early-life post-hatching^a, b^.

**Met**	−		+		***P*-value**		
**DS**	**−**	**+**	**−**	**+**	**Met**	**DS**	**Met × DS**
Maltase activity in jejunum, μmol·min^−1^·g^−1^ tissue							
Day of hatching	3.38 ± 0.34	4.51 ± 0.66	3.52 ± 0.49	4.44 ± 0.89	0.898	0.001	0.694
Day 7 post-hatching	7.00 ± 0.14	7.66 ± 0.69	7.57 ± 0.65	7.65 ± 0.39	0.202	0.093	0.184
Day 28 post-hatching	5.17 ± 0.66	5.29 ± 0.61	5.69 ± 0.71	5.76 ± 0.73	0.087	0.724	0.939
Sucrase activity in jejunum, μmol·min^−1^·g^−1^ tissue							
Day of hatching	1.06 ± 0.16	1.80 ± 0.16	1.09 ± 0.22	1.64 ± 0.39	0.554	<0.001	0.380
Day 7 post-hatching	3.29 ± 0.26^c^	4.24 ± 0.24^ab^	4.41 ± 0.08^a^	4.16 ± 0.07^b^	<0.001	<0.001	<0.001
Day 28 post-hatching	4.26 ± 0.18	4.59 ± 0.52	4.39 ± 1.23	4.85 ± 0.43	0.520	0.186	0.829
Na^+^/K^+^ATPase activity in jejunum, U·min^−1^·g^−1^ tissue							
Day of hatching	5.71 ± 0.20	6.71 ± 0.84	6.58 ± 1.19	7.38 ± 0.98	0.046	0.021	0.773
Day 7 post-hatching	21.84 ± 0.72	20.97 ± 1.17	20.68 ± 2.91	20.10 ± 1.41	0.173	0.324	0.843
Day 28 post-hatching	22.27 ± 0.56	22.90 ± 1.77	22.07 ± 1.90	23.17 ± 1.03	0.959	0.153	0.697
Alkaline phosphatase activity in jejunum, μmol·min^−1^·g^−1^ tissue							
Day of hatching	1.15 ± 0.10^d^	1.39 ± 0.16^c^	1.64 ± 0.10^b^	2.74 ± 0.27^a^	<0.001	<0.001	<0.001
Day 7 post-hatching	8.44 ± 0.75	10.38 ± 1.85	9.80 ± 1.31	12.75 ± 1.84	0.007	0.001	0.425
Day 28 post-hatching	11.96 ± 2.39	11.68 ± 2.35	9.99 ± 0.65	12.36 ± 1.32	0.404	0.178	0.092

^1^The data are presented as the means ± standard deviation.

^2^The “−” means without nutrients injection, while “+” means with nutrients injection.

a, b, c, d Different superscripts within a row indicate a significant difference (*p* < 0.05).

### Jejunum antioxidant parameters

The administration of Met increased GSH (*P* < 0.01) and GPX (*P* < .01) levels, whereas decreased GSSG (*P* < 0.01) levels and GSSG/GSH (*P* < 0.01) ratio from jejunum on the day of hatching. In addition, the GPX levels on days 7 (*P* < 0.01) and 28 (*P* < 0.01) post-hatching was increased by Met injection. However, DS injection had no significant effects on the jejunum antioxidant parameters (Table 11).

**Table 11:** Effects of *in ovo* injection of methionine (Met) and disaccharide (DS) on jejunum antioxidant parameters of geese during the early-life post-hatching^a, b^.

**Met**	−		+		***P*-value**		
**DS**	**−**	**+**	**−**	**+**	**Met**	**DS**	**Met × DS**
GSSG, μmol·g^−1^ tissue							
Day of hatching	7.16 ± 0.63	6.95 ± 0.60	6.31 ± 0.24	6.30 ± 0.25	0.001	0.585	0.618
Day 7 post-hatching	10.86 ± 0.87	10.89 ± 0.88	11.15 ± 0.76	10.95 ± 0.28	0.576	0.776	0.706
Day 28 post-hatching	4.93 ± 0.59	4.98 ± 0.35	4.89 ± 0.51	4.75 ± 0.36	0.481	0.816	0.620
GSH, μmol·g^−1^ tissue							
Day of hatching	181.23 ± 14.01	179.92 ± 14.72	223.61 ± 19.68	221.67 ± 25.75	<0.001	0.837	0.968
Day 7 post-hatching	265.83 ± 24.37	262.56 ± 25.93	278.78 ± 20.61	285.01 ± 13.86	0.059	0.869	0.597
Day 28 post-hatching	161.04 ± 18.83	159.63 ± 11.97	162.52 ± 12.63	164.19 ± 10.23	0.598	0.981	0.786
GSSG/GSH							
Day of hatching	0.040 ± 0.01	0.039 ± 0.01	0.028 ± 0.002	0.029 ± 0.003	<0.001	0.891	0.774
Day 7 post-hatching	0.041 ± 0.01	0.042 ± 0.001	0.040 ± 0.003	0.038 ± 0.002	0.155	0.658	0.512
Day 28 post-hatching	0.031 ± 0.01	0.031 ± 0.004	0.030 ± 0.001	0.029 ± 0.002	0.256	0.829	0.624
GPX, U·g^−1^ tissue × 10^3^							
Day of hatching	10.90 ± 0.46	11.20 ± 0.90	13.25 ± 1.23	12.83 ± 1.27	<0.001	0.877	0.395
Day 7 post-hatching	7.32 ± 0.85	7.38 ± 0.79	8.54 ± 0.79	8.54 ± 0.89	0.002	0.924	0.924
Day 28 post-hatching	7.28 ± 0.67	7.34 ± 0.83	8.72 ± 0.92	8.18 ± 0.44	0.001	0.427	0.328

GSH, glutathione; GPX, glutathione peroxidase; GSSG, glutathione disulfide.

a The data are presented as the means ± standard deviation.

b The “−” means without nutrients injection, while “+” means with nutrients injection.

### Jejunum nutrients transport gene expression

Effects of *in ovo* injection of Met and/or DS on jejunum nutrients transport gene expression were shown in Table 12. The Met injection upregulated the expression of GLUT-2 on the day of hatching (*P* < 0.05) and day 28 post-hatching (*P* < 0.05). DS injection upregulate the SGLT-1 expression on the day of hatching (*P* < 0.01) and day 7 post-hatching (*P* < 0.01), GLUT-2 expression on the day of hatching (*P* < 0.01), day 7 post-hatching (*P* < 0.01), and day 28 post-hatching (*P* < 0.01), and SI expression on the day of hatching (*P* < 0.01), day 7 post-hatching (*P* < 0.01), and day 28 post-hatching (*P* < 0.01). Additionally, the expression of GLUT-2 was synergistically upregulated by *in ovo* injection of Met plus DS on day 28 post-hatching (*P* < 0.01).

**Table 12:** Effects of *in ovo* injection of methionine (Met) and disaccharide (DS) on jejunum nutrients transport gene expression of geese during the early-life post-hatching^1, 2^.

**Met**	−		+		***P*-value**		
**DS**	**−**	**+**	**−**	**+**	**Met**	**DS**	**Met × DS**
SGLT-1 expression abundance in jejunum							
Day of hatching	1.00 ± 0.13	1.80 ± 0.19	1.06 ± 0.08	1.86 ± 0.12	0.248	<0.001	0.978
Day 7 post-hatching	5.82 ± 0.74	9.83 ± 1.09	5.45 ± 0.77	10.31 ± 0.99	0.881	<0.001	0.267
Day 28 post-hatching	5.23 ± 0.92	5.50 ± 0.47	5.13 ± 0.72	5.49 ± 0.46	0.832	0.262	0.871
GLUT-2 expression abundance in jejunum							
Day of hatching	1.00 ± 0.14	1.44 ± 0.10	1.14 ± 0.12	1.56 ± 0.20	0.037	<0.001	0.861
Day 7 post-hatching	5.20 ± 0.64	8.05 ± 0.79	5.65 ± 0.62	8.09 ± 0.84	0.436	<0.001	0.497
Day 28 post-hatching	1.37 ± 0.20^c^	3.66 ± 0.32^b^	1.40 ± 0.29^c^	4.62 ± 0.82^a^	0.019	<0.001	0.025
SI expression abundance in jejunum							
Day of hatching	1.00 ± 0.15	1.58 ± 0.19	1.14 ± 0.08	1.57 ± 0.13	0.281	<0.001	0.208
Day 7 post-hatching	3.44 ± 0.35	5.35 ± 0.65	3.06 ± 0.24	5.46 ± 1.26	0.654	<0.001	0.424
Day 28 post-hatching	1.02 ± 0.09	1.91 ± 0.23	1.08 ± 0.14	2.07 ± 0.12	0.101	<0.001	0.454

SGLT-1, sodium/glucose cotransporter protein-1; GLUT-2. glucose transporter-2; SI, sucrase-isomaltase.

1 The data are presented as the means ± standard deviation.

2 The “−” means without nutrients injection, while “+” means with nutrients injection.

a, b, c Different superscripts within a row indicate a significant difference (*p* < 0.05).

## Discussion

In the present study, the birth weight of goslings was improved by feeding DS into the embryo. Similarly, Foye et al. (23) noted that *in ovo* injection of carbohydrates (20% dextrin and 3% maltose dissolved in 0.9% saline) improved the growth performance of birds, manifested in high body weight. Indeed, the delivery of carbohydrates into the embryo has been reported to increase the level of available energy, thus avoiding the reduction of body weight induced by the lack of energy (24). We also observed the increase in liver, breast muscle, and total glycogen contents induced by DS delivery on the day of hatching, which indicated an increase in the level of available energy in the embryo. Therefore, we concluded that *in ovo* injection of DS positively affects the birth weight of goslings, which was attributed to the enhancement of energy storage.

The breast muscle is the largest tissue in the body of poultry, and it plays an important role in metabolic activity because of its relatively large size and glycogen storage. The development of skeletal muscle is a complex process, which comprises the differentiation of myoblast from the mesodermal precursor cells to mature myotubes, thus differentiating to form myofibers during embryogenesis (25–27). Early feeding has been reported to be a suitable strategy to promote the maturation of myofiber, which leads to myofibers with larger diameters (1). Uni et al. (28) injected the DS (25 g/L maltose, 25 g/L sucrose, 200 g/L dextrin, and 1 g/L β-hydroxy-β-methylbutyrate dissolved in 5 g/L NaCl) into the amnion of a chicken egg and found a higher growth of breast muscle. Dong et al. (29) noted that *in ovo* feeding of DS (25 g/L maltose and 25 g/L sucrose dissolved in 7.5 g/L saline) had stimulating effects on the development of breast muscle in pigeons. In this study, we also observed that *in ovo* injection of DS increased the relative weight of breast muscle and diameter of myofiber in geese. Additionally, the relative weight of breast muscle and diameter of myofiber were also improved in Met injection. However, studies on the effects of *in ovo* feeding of Met on the breast muscle parameters were still limited. Mechanisms in Met administration that promote the development of breast muscle may be related to sarcoplasmic hypertrophy (30, 31). Sarcoplasmic hypertrophy would lead to the increase of myofiber cross-sectional area (32). According to Sahebi-Ala et al. (33), Met as a donor of the sulfur group was beneficial to enhancing the diameter of myofiber, resulting in the development of muscle. Therefore, we concluded that *in ovo* injection of Met or DS had positive effects on the development of breast muscle. However, either Met or DS, the improvement effect of nutrient delivery on breast muscle parameters was only observed in the early-life post-hatching of gosling, while not observed on day 28 post-hatching, which indicated that the injection of DS or Met have short-term effect on the improvement of breast muscle.

Additionally, the growth and development of muscle are usually regulated by specific genes and transcription factors which include Myf-5 and MSTN. Myf-5 is a myogenic regulatory factor and its expression is beneficial to the development of muscle fiber (34, 35). MSTN also known as a growth factor and differentiation factor-8 is mainly expressed in skeletal muscle cells and acts as an essential negative regulator in the growth of skeletal muscle in poultry (36, 37). The downregulation of MSTN expression is contributed to the hypertrophy of myofiber and the development of breast muscle (38). In this study, DS injection upregulated the expression of Myf-5 on the day of hatching, which was matched to the improvement of breast muscle parameters. Additionally, the improvement effects of Met administration on the upregulation of Myf-5 expression and the downregulation of MSTN expression were observed in each measured timepoint. However, higher breast muscle development did not last until day 28 post-hatching. An investigation of the development situation of other muscle parts is needed. As described by Wang et al. (39), the muscle growth-related genes have different expression schedules in different tissues (legs and breast muscle). In brief, we concluded that *in ovo* delivery of DS or Met positively affects the development of breast muscle by regulating the expression of breast muscle growth-related genes during the early-life post-hatching of goslings.

On the other hand, *in ovo* injection technique has been reported to support muscle growth *via* enhancing the liver and muscle glycogen reserves during post-hatching periods (1). Insufficient glycogen reserves will force the embryo to mobilize more muscle proteins toward gluconeogenesis, thus restricting the development of breast muscle (28). A sufficient glycogen store reduces the need for glucose synthesis *via* gluconeogenesis from muscle protein (3), resulting in the development of breast muscle (28). It has been reported that *in ovo* injection of carbohydrates improved embryo energy reserves status (10). Uni et al. (28) reported that *in ovo* injection of DS (25 g/L maltose, 25 g/L sucrose, 200 g/L dextrin, and 1 g/L β-hydroxy-β-methylbutyrate dissolved in 5 g/L NaCl) increased liver and muscular glycogen reserves in chicks. Foye et al. (23) noted that DS (20% dextrin and 3% maltose dissolved in 0.9% saline) injection increased the liver and muscle glycogen levels in turkey. In the present study, we also observed that DS injection increased the liver and muscle glycogen contents, which indicated an enhancement in glycogen store. Additionally, glycogen reserves have been reported to be negatively correlated with G-6-Pase activity (40). G-6-Pase is a membrane-bound enzyme, which is located on the internal membrane of the endoplasmic reticulum. It plays a key role in the terminal step of gluconeogenesis and glycogenolysis, and it can catalyze the conversion of glucose-6-phosphate to glucose, thus releasing glucose from the cell (41, 42). In the present study, DS injection inhibited the G-6-Pase activity. However, Met injection had no significant effects on the glycogen reserves and G-6-Pase activity. We concluded that the promotion effect of DS injection on the development of breast muscle was partially attributed to the increase of glycogen reserves induced by reducing G-6-Pase activity. However, it is worth noticing that the enhancement of glycogen reserves and the reduction of G-6-Pase activity induced by DS administration synchronously occurred, and only presented in the early life of goslings, which indicated the important role of G-6-Pase in regulating the glycogen reserves in the early life and the effect is temporary.

As reported, the increased growth of “demand” tissue such as skeletal muscle should be supported by the development in structure and function of “support” tissue such as gastrointestinal tract (43). Early growth and development of the gastrointestinal tract are critical to optimizing the growth of poultry. The greatest nutrient digestion and absorption happens in the small intestine of poultry, especially the jejunum (44). In the small intestine, the epithelium is thrown into long folds, the villi, which serves to increase the surface area for enzyme secretion and nutrient absorption. Histomorphology is one of the most commonly used parameters to evaluate the status of the gut. Generally, the length and relative weight of the small intestine, as well as the surface area, width, and height of villi from the small intestine, are considered important factors that reflect the development of the small intestine. It has been reported that *in ovo* feeding of maltose promoted the development of the jejunum villi in poultry (45). Chen et al. (46) noted that DS (25 g/L maltose and 25 g/L sucrose dissolved in 4.5 g/L NaCl) injected into the amnion of duck embryo improved the length and surface area of jejunal villi. In this study, we also observed that *in ovo* injection of DS increased jejunal villus height and surface area. Additionally, Met also has been reported to be crucial for the maintenance of gut integrity and function (47). Mohammadrezaei et al. (48) reported that *in ovo* injection of Met enhanced the nutrients absorption ability of chicks by altering the height and width of villi from the small intestine. Chen et al. (49) noted that Met injection increased the relative weight of the duodenum, jejunum, and ileum, as well as the villus height in the small intestine of chicks. Similarly, in the present study, DS injection provisionally improved the development of jejunal villi but did not affect the small intestine parameters. However, Met administration has a long-term effect on the improvement of jejunal villi development and a short-term effect on the improvement of small intestine parameters. Therefore, we concluded that *in ovo* delivery of Met or DS promoted the development of jejunal villi and small intestine in the early life, which was beneficial to support the absorption of the nutrients.

A mature intestine is often accompanied by abundant digestive enzyme secretion (46). The digestive enzyme plays an important role in the digestion of nutrients into smaller nutrient molecules to facilitate the absorption by the host. It has been reported that a high disaccharidase activity presented in the small intestine ensures rapid carbohydrate digestion, and it breaks down the DS into glucose (50). The alkaline phosphatase involves in digestive processes such as the absorption of glucose (51). Chen et al. (46) reported that DS (25 g/L maltose and 25 g/L sucrose dissolved in 4.5 g/L NaCl) injection increased jejunal sucrase activity in ducks. Similar to the above study, *in ovo* delivery of Met or DS increased the activities of digestive enzymes in the jejunum; however, this improvement effect was temporary. Further investigation is needed to evaluate the effects of *in ovo* Met or DS delivery on the digestive enzyme activities. We concluded that *in ovo* administration of Met or DS had positive effects on the activities of digestive enzymes in jejunum during the early life of goslings.

The nutrient absorption in the intestine is relying on the Na^+^-dependent kinetics (52). Sodium transportation is achieved by the enterocyte's basolateral Na^+^/K^+^ATPase (52). Na^+^/K^+^ATPase pumps out sodium to cells whereas it pumps potassium into cells, which is beneficial to maintaining resting potential and regulating nutrient transportation (53–55). In this study, we observed that *in ovo* injection of Met or DS improved the Na^+^/K^+^ATPase activity in the jejunum in early life, with no long-term effect, which indicated that *in ovo* delivery strategies used in this study had positive effects on the nutrient transportation in the small intestine during the early life.

Moreover, the transport mediators expressed in the apical and basal membrane of enterocytes also play a key role in nutrient absorption. Nutrients are transported into enterocytes by special transporters. The expression of the nutrient transport gene could be used as an indicator of the growth and absorptive capacity of the small intestine (56). Because very small amounts of carbohydrates are presented in the intestine during the late term of incubation, the increase in SI expression at the apical membrane allows for the degradation of carbohydrates into glucose (57). Maintaining high expression levels of SI in the small intestine provides a sufficient supply of substrates for the nutrient transporters such as SGLT-1 and GLUT-2 (57). Glucose is the key fuel and metabolic substrate that could be absorbed *via* SGLT-1, which is located on the apically of the intestinal epithelium, and transported into the blood *via* GLUT-2, which is expressed on the basolateral membrane (58, 59). In this study, *in ovo* injection of Met long-term upregulated the expression of GLUT-2, while DS injection short-term upregulated the expression of SGLT-1, long-term upregulated the expression of GLUT-2, and downregulated the expression of SI. Similarly, Dong et al. (29) reported that the enhancement of the jejunal SGLT-1 and GLUT-2 expression was found during post-hatching periods in pigeon embryos that received carbohydrate solution (2.5% maltose and 2.5% sucrose dissolved in 0.75% saline) into the amniotic fluid. On the other hand, SGLT-1 has been reported to be coupled to the action of the Na^+^/K^+^ATPase to mediate glucose transportation (59, 60). GLUT-2 has been reported to be combined with G-6-Pase acted in concert to control the release of glucose from the liver (61). In this study, the increase of Na^+^/K^+^ATPase activity was observed by *in ovo* injection of DS or Met, moreover, *in ovo* injection of DS decreased G-6-Pase activity. Therefore, we concluded that *in ovo* injection of DS and/or Met improved nutrient absorption by regulating the nutrient transport gene expression and digestive enzyme activities.

It has been reported that the expression of GLUT-2 was inhibited by the stress response in poultry (62). However, this inhibition effect could be reversed by alleviating oxidative stress (63). Ibrahim et al. (64) reported that feeding broiler chicks with antioxidant herbal extract improved the antioxidant situation of birds, thus upregulating the expression of GLUT-2. The decrease of GSSG and the increase of GSH and GPX concentrations are characteristic of the alleviation of oxidative stress (65). Met is known to decrease oxidative stress and has antioxidant properties (66). Its *in ovo* delivery has been reported to improve the antioxidative capacity in the embryo (66). In this study, Met injection short-term increased GSH concentration and decreased GSSG and GSSG/GSH levels from jejunum, as well as long-term increased jejunal GPX concentration. Therefore, we concluded that *in ovo* delivery of Met is beneficial to improve the antioxidative situation of jejunum, thus improving the GLUT-2 expression and further improving intestinal health. Abolfathi et al. (67) reported that feeding broiler chicks with antioxidant herbal extract improved intestinal morphology through increasing intestinal antioxidant capacity. Long et al. (68) noted that dietary supplementation of antioxidant substances improved intestinal antioxidant capacity, thus increasing intestinal digestive enzyme activities. Therefore, we considered that the improvement of jejunum antioxidant parameters by Met injection also benefited the improvement of intestine morphology and digestive enzyme activities.

## Conclusion

Our results demonstrated that DS injection increased glycogen reserves and regulated muscle growth-related gene expression, thus improving breast muscle parameters and birth weight during the early-life post-hatching. The short-term effect on the improvement of digestive enzyme activities, nutrient transport enzyme activities, and jejunal villus parameters, as well as the long-term effect on the regulation of nutrient transport-related gene expression, were observed to deliver DS into the embryo, which contributed to promoting the nutrient absorption in post-hatching.

*In ovo* injection of Met continuously regulated the breast muscle growth-related gene expression, thus improving breast muscle parameters. The temporary improvement effect of Met delivery on the digestive enzymes activities, nutrient transport enzymes activities, small intestine parameters, and nutrient transport-related gene expression, as well as the continuous improvement effect on the jejunal villus parameters and jejunal antioxidative situation, were beneficial to support the nutrient absorption after incubation.

Moreover, *in ovo* feeding of DS plus Met mixture synergistically improved the diameter of myofiber, jejunal villus height and width, jejunal sucrase, and alkaline phosphatase activities in early-life post-hatching, but continuously upregulated the expression of jejunal GLUT-2. Therefore, we concluded that *in ovo* delivery of DS plus Met is a suitable strategy to improve the nutrient absorption and breast muscle development of goslings post-hatching.

## Data availability statement

The raw data supporting the conclusions of this article will be made available by the authors, without undue reservation.

## Ethics statement

The animal study was reviewed and approved by the Animal Care and Use Committee of Jilin Agricultural University (Changchun, China).

## Author contributions

DD: writing—original draft, investigation, and writing—review and editing. HZ: formal analysis and investigation. YL: conceptualization and methodology. DL: supervision and writing—review and editing. All authors contributed to the article and approved the submitted version.

## Conflict of interest

The authors declare that the research was conducted in the absence of any commercial or financial relationships that could be construed as a potential conflict of interest.

## Publisher's note

All claims expressed in this article are solely those of the authors and do not necessarily represent those of their affiliated organizations, or those of the publisher, the editors and the reviewers. Any product that may be evaluated in this article, or claim that may be made by its manufacturer, is not guaranteed or endorsed by the publisher.
